# Highly efficient and durable antimicrobial nanocomposite textiles

**DOI:** 10.1038/s41598-022-22370-2

**Published:** 2022-10-15

**Authors:** Vinni Thekkudan Novi, Andrew Gonzalez, John Brockgreitens, Abdennour Abbas

**Affiliations:** 1 Department of Bioproducts and Biosystems Engineering, University of Minnesota Twin Cities, 2004 Folwell Ave, St. Paul, MN 55108 USA; 2 Claros Technologies Inc., 1600 Broadway St NE, Suite 100, Minneapolis, MN 55413 USA

**Keywords:** Microbiology, Health care, Chemistry, Engineering, Materials science, Nanoscience and technology

## Abstract

Healthcare associated infections cause millions of hospitalizations and cost billions of dollars every year. A potential solution to address this problem is to develop antimicrobial textile for healthcare fabrics (hospital bedding, gowns, lab coats, etc.). Metal nanoparticle-coated textile has been proven to possess antimicrobial properties but have not been adopted by healthcare facilities due to risks of leaching and subsequent loss of function, toxicity, and environmental pollution. This work presents the development and testing of antimicrobial zinc nanocomposite textiles, fabricated using a novel Crescoating process. In this process, zinc nanoparticles are grown in situ within the bulk of different natural and synthetic fabrics to form safe and durable nanocomposites. The zinc nanocomposite textiles show unprecedented microbial reduction of 99.99% (4 log_10_) to 99.9999% (6 log_10_) within 24 h on the most common Gram-positive and Gram-negative bacteria, and fungal pathogens. Furthermore, the antimicrobial activity remains intact even after 100 laundry cycles, demonstrating the high longevity and durability of the textile. Independent dermatological evaluation confirmed that the novel textile is non-irritating and hypoallergenic.

## Introduction

Healthcare associated infections (HAIs) are of major public health concern with at least one in 31 patients getting infected during or after receiving treatment in hospitals in the United States^1^. Textiles in healthcare facilities (curtains, bedding, workers’ clothing, carpets, patient gowns, towels, furniture) are known to harbor microorganisms and facilitate the spread of HAIs including SARS-CoV-2^2,3^. Periodic cleaning and applying disinfectants to all textiles in the hospital environment is not sufficient to prevent transmission. Even with regular cleaning, healthcare workers’ clothing was found to have significant amount of microbial load after a typical work schedule of 8–12 h, and about 92% of hospital curtains contained pathogens within a week after cleaning^4^.

The magnitude of this problem has further increased since the COVID-19 pandemic that kept healthcare facilities at maximum capacity and prone to the spread of more infections. Among the common HAIs seen in hospitals in the United States, central line-associated bloodstream infection (CLABSI), catheter-associated urinary tract infections (CAUTI) and ventilator-associated pneumonia increased by 47%, 19% and 45%, respectively, in the year 2020. Apart from these, the Centers for Disease Control and Prevention (CDC) also reported that infections associated with methicillin resistant *Staphylococcus aureus* (MRSA) increased in the year 2020, with the onset of the COVID-19 pandemic^5^.

In the United States, transmission of SARS-CoV-2 has been predominant in hospitals, nursing homes and medical facilities resulting in over 1037,400 healthcare workers contracting COVID-19 in the US as of March 18, 2022^6,7^. While the focus of healthcare workers has shifted to combating this problem, some of the leading causes of nursing home deaths have taken a backseat, such as respiratory infections, urinary tract infections (UTIs), gastroenteritis, sepsis and skin diseases, which involve multidrug resistant bacterial and fungal pathogens^8,9^. This makes these facilities “hotspots” for such infections resulting in an urgent need for self-disinfecting antiviral/antimicrobial textiles that curb transmission where ideally low capital investment is required.

The use of nanotechnology and nanomaterials is one of the most promising approaches for the development of the next generation of functional textiles^10,11^. The importance of nanomaterials, particularly nanoparticles, lies in their ability to confer multiple functionalities with remarkable enhancement of these functionalities due to increased surface-to-volume ratio and high surface energy^12^. Silver nanoparticles in particular have seen growing interest from the textile industry due to their wide-spectrum antimicrobial properties. Between 2004 and 2011, the silver market share of antimicrobial textiles increased from 9 to 25%, progressively replacing synthetic organic compounds^13^. Several methods have been developed to incorporate metal and metal-oxide nanoparticles into textiles^14^. The most common approaches include spraying of a nanoparticle solution^15^, layer-by-layer deposition with polymers or polyelectrolytes^16^, sonochemical coating^17^, plasma deposition^18^, and electrospinning^19^.

Current methods of nanoparticle functionalization have significant limitations in terms of long-term durability^20^. Nanoparticle surface coatings are subject to leaching as nanoparticles are released from the fiber material. This is particularly pertinent in textiles and clothing that are subject to mechanical strain, abrasion as well as repeated wash and dry cycles. Silver nanoparticle emission from textiles varies among products and can be as high as 80% during the first wash^20,21^. Furthermore, silver, and other nanoparticles utilized in textiles such as copper, have demonstrated toxicity in environmental systems^22–24^. These antimicrobial nanoparticles can also disrupt biological treatment processes used in municipal and industrial water treatment facilities^25^.

The work presented here focuses on the use of a novel process called Crescoating, meaning coating by growth [-cresco], for the introduction of nanoparticles into textile to form stable nanocomposites and the demonstration of the antimicrobial effect of zinc nanocomposite textiles. Typically, nanocomposites are described as at least two-phased materials with one of the components being in the nanoscale. This could either be a metal or a non-metal nanoparticle phase embedded in a macroscale support such as the fabrics in this case. In the Crescoating treatment process nanoparticles are grown directly on and within the bulk of polymeric materials. This bulk growth enables improved nanoparticle retention, preventing both textile performance losses and nanoparticle emissions into water systems.

Zinc has been selected due to its numerous advantages over nanoparticles currently used in textiles. Besides its antimicrobial properties^26^ it is Generally Recognized as Safe (GRAS) by the US Food and Drug Administration (FDA), does not exhibit environmental or human toxicity, and is commonly used in commercial products ranging from food to cosmetics^26^. The demonstration of the antimicrobial properties was performed by testing different textiles against different microorganisms including Gram-negative, Gram-positive bacteria, and fungi.

## Results and discussion

### Textile zinc nanocomposite synthesis

**Figure 1:**
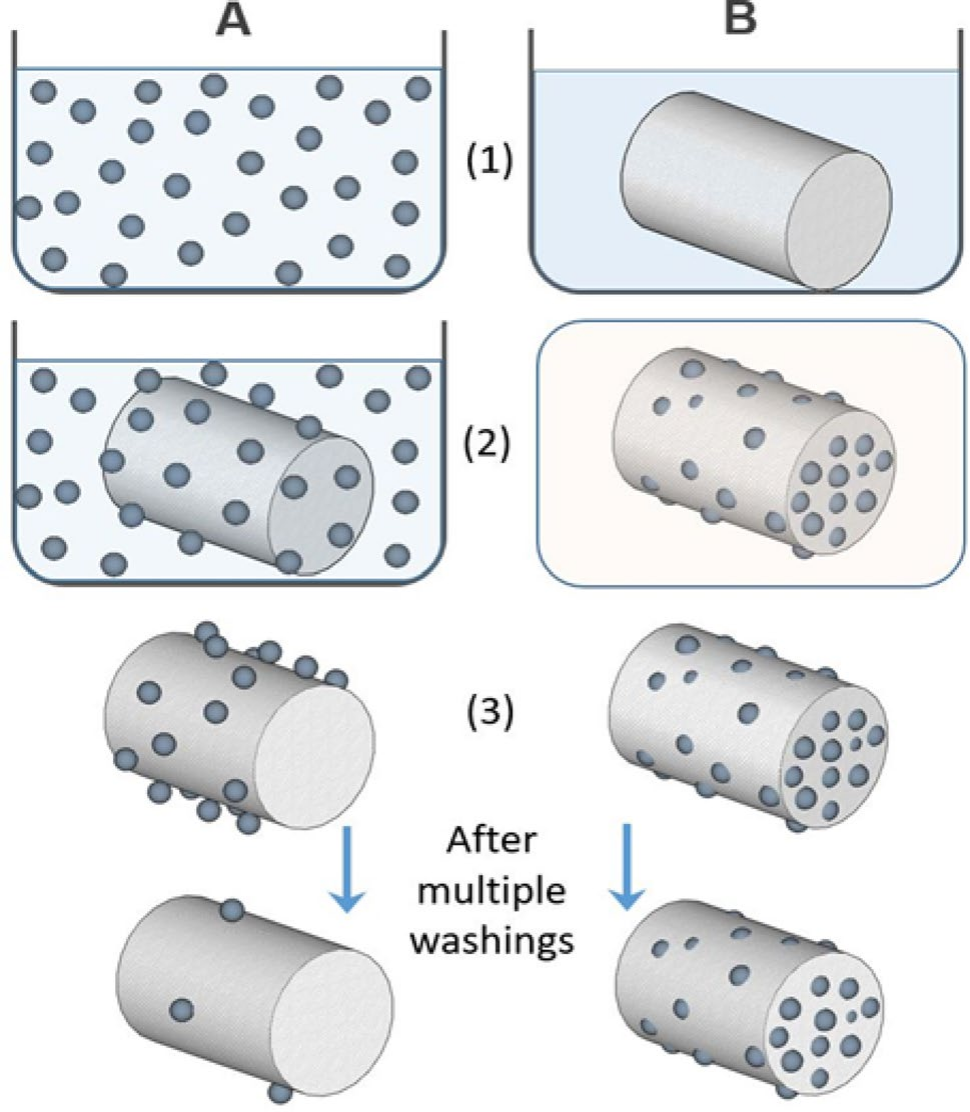
Comparison of (**A**) conventional dip-coating process with (**B**) thermal Crescoating technology. (**A**) Wet synthesis of nanoparticles by chemical reduction (1), dip-coating of the textile in the nanoparticle (2), followed by washing and drying (3). (**B**) Impregnation of the textile in precursor solution (1), Thermal reduction by heating the textile at 100 °C (2), followed by washing and drying (3).

The nanocomposite textiles were fabricated by soaking the textiles in an aqueous ionic precursor solution, which is thermally treated to form nanoparticles in the bulk and throughout the surface of the fabrics. The technology used here is called Crescoating (Fig. 1) and begins with solid seed formation from the ionic solution under heating followed by their nucleation to form defined nanoparticles. The thermal treatment was done using a convection oven which leads to the evaporation of water followed by seed formation and nucleation of the particles. The size of the particles could range from 5 to 500 nm, depending on the process conditions and they get embedded in the textile supports. This method improves the durability and longevity of the nanocomposite textiles, unlike conventional surface-coated products, which lose their nanoparticles over time due to multiple washing cycles.

**Figure 2:**
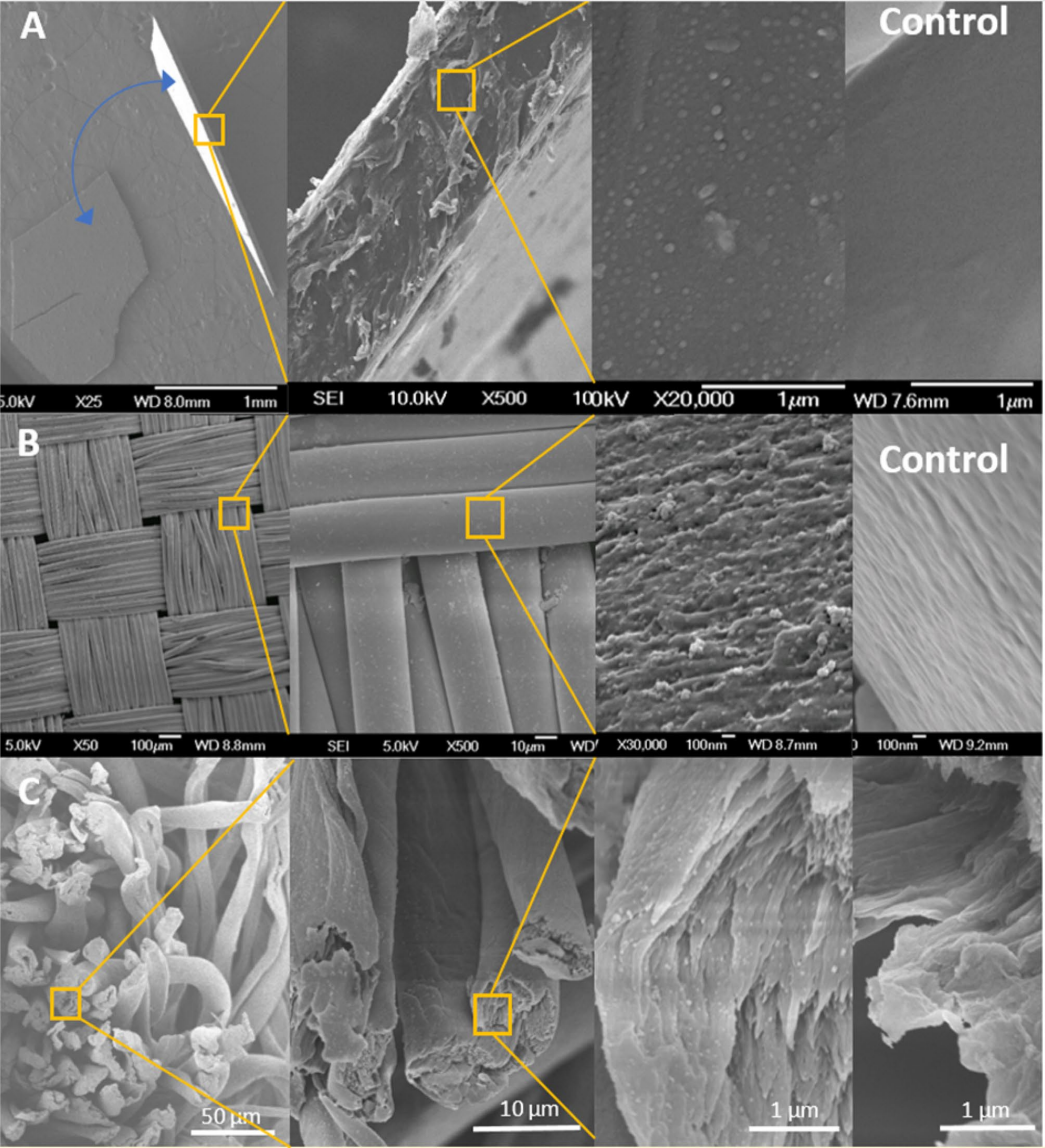
SEM images of plastic nanocomposites produced with “in situ growth” process. (**A**) Zinc-polyurethane nanocomposite film. The blue arrows show two pieces of the nanocomposite thin film. Image amplification at the film cross-section shows the presence of zinc nanoparticles inside the film. (**B**) Zinc-nylon nanocomposite showing zinc nanoparticles embedded with the nylon fibers. (**C**) Silver-polyester/cotton nanocomposite.

This method’s efficacy was previously demonstrated for environmental remediation using polyurethane foam, and polypropylene, polycotton and nylon cotton fabrics for their antiviral properties^27–30^. Figure 2 shows SEM images of nanocomposite polyurethane, nylon and polyester materials.

**Figure 3:**
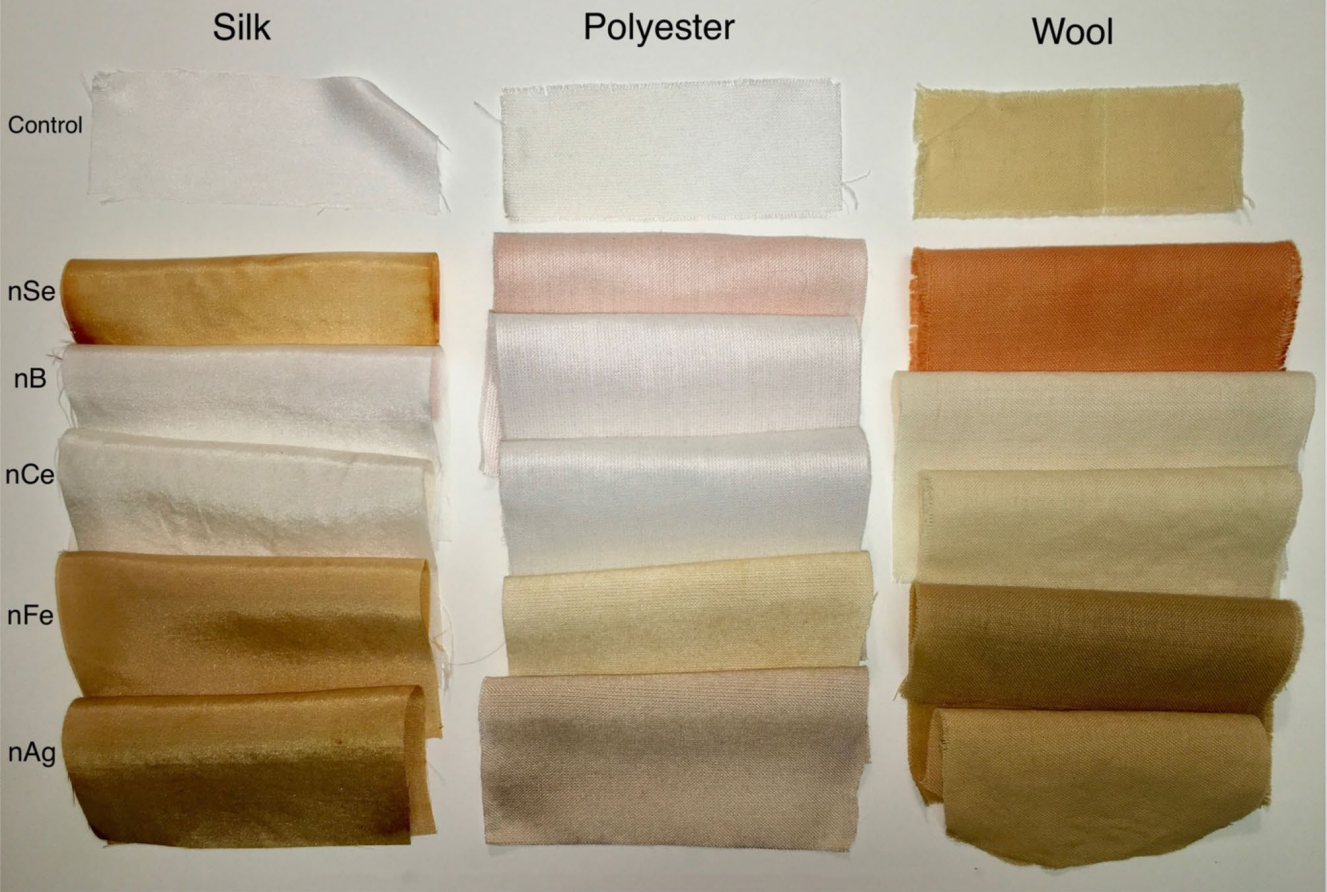
Versatility of the in situ nanoparticle growth process using different nanoparticles on different textiles. nSe: nanoselenium, nb: nanoboron, nCe: nanocerium, nFe: nanoiron, nAg, nanosilver.

To demonstrate the versatility of the Crescoating technology the process has been adapted to various fabrics using different metal nanoparticles as well (Fig. 3).

In this study the fabric supports used were silk, synthetic polyester, nylon cotton and polyester cotton. Natural, synthetic and blended textiles were selected to analyze how well the antimicrobial treatment would work on different types of fabrics. Specifically, the above chosen set of fabrics are commonly used in the consumer apparel and textile industry and incorporating antimicrobial activity in them would be beneficial. The synthesis of zinc nanocomposite textiles was based on a previously developed method^30^. Briefly, the fabrics were initially submerged in precursor ionic solutions of zinc salt for 30 min at room temperature. They were then heated in a convection oven at 100 °C for 4 h by maintaining a thin layer of the solution over them. White precipitates were formed on the zinc supports. Any unbound precipitate was washed away by following the American Association of Textile Chemists and Colorists (AATCC) LP1: Home Laundering method and dried^31^.

#### Characterization

##### Structure and characterization:

**Figure 4:**
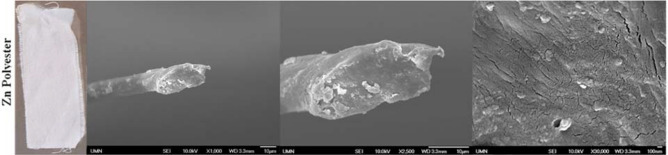
SEM images of nanoparticles harvested from zinc treated polyester fabric.

The synthesized nanocomposites were characterized using scanning electron microscopy (6700 SEM, JEOL Inc.). Figure 4 depicts zinc nanocomposites grown in polyester fabrics. Very small zinc nanoparticles in a size range of ≤ 100 nm can be seen on the textile support. Nanoparticle coverage on the support is not uniform nor widespread. SEM images of untreated control fabrics can be found in Supplementary Figure S1. Unbound nanoparticles collected through hand washing the nanocomposite fabrics immediately after synthesis were analyzed using x-ray crystallography (D8 Discover, Bruker Corp.) in our previous study^30^. Pattern fitting was conducted with JADE for XRD software (Materials Data Inc.). Zinc particles were primarily comprised of two crystalline phases of zinc hyroxide (Zn(OH)_2_) and zinc carbonate hydroxide (Zn_5_(CO_3_)_2_(OH)_6_ hydrozincite).

The formation of these nanoparticles follows a three step process similar to the formation of iron oxide nanoparticles previously studied^28^. Thermal synthesis of supported nanoparticles is achieved through the formation of solid phase seeds that nucleate to form nanoparticles. For zinc, the salt precursors undergo hydrolysis to form hydroxide intermediates in the form of Zn(OH)_2_. In the presence of carbonate contributed from the atmosphere and/or from the degradation of the acetate counterion, zinc carbonate hydroxide particles form. The hydrozincite and zinc hydroxide phases can be seen in x-ray diffraction patterns^30,32^.

##### Synthetic precipitate leachate procedure

The retention of zinc nanoparticles in the textiles after several wash cycles was tested by a third party testing company, Pace Analytical LLC. Samples of several types of nanocomposite cotton fabrics were subjected to the synthetic precipitation leachate procedure (SPLP). These fabrics were previously washed once post manufacturing to remove loose nanoparticles from their surface. The test based on the EPA 3010A preparation and EPA 6010B analytical method was then applied to study the leaching of zinc nanoparticles from the fabrics^33^. The results from the study showed that the highest zinc leaching of 106,000 µg/L occurs after the first post-fabrication wash for one of the cotton types. All other values are lower and are followed by significantly lower leaching after every subsequent wash cycle (See supplementary information Table S1). This shows that the nanocomposite fabrics prepared using the novel Crescoating method are highly durable. The highest initial leachate concentration is found to be much lower than 250,000 µg/L, which is the soluble threshold limit concentration (STLC) value for California state^34^. Based on this limit it can be concluded that the concentration of zinc leaching from the fabrics after each laundering cycle is not significant enough to pose a health or environmental hazard. The different values of leaching observed for the three different cotton types could be a result of differences in their fiber sizes and pretreatment processes such as mercerization (oxidization of fibers for metal easier metal binding), which was done for cotton types 1 and 2.

### Antimicrobial application test

For antimicrobial testing, zinc nanoparticles were grown on polyester, silk and nylon/cotton (50:50) textile swatches obtained from Testfabrics Inc. and Rockywoods Fabrics LLC, respectively. AATCC Test Method 100–2004 was used for antibacterial and antifungal testing of functionalized fabrics. The experiment was done in triplicates. Two bacterial species *Pseudomonas aeruginosa* (PA, ATCC 27853) (Gram-negative) and methicillin resistant *Staphylococcus aureus* (MRSA) (Gram-positive) and a fungal species *Candida albicans* (CA) were selected for antimicrobial testing. The results have been separated into two categories: a “before washing” test conducted on swatches that have been machine washed soon after treatment and an “after washing” test with swatches that were subjected to more machine washing and drying cycles. The “after washing” tests were conducted by a third-party testing company (See report in supplementary information). The fabric used was polyester cotton blend and the tests were conducted on the bacterial species *Staphylococcus aureus* (SA, ATCC 6538) (Gram-positive) and *Klebsiella pneumoniae* (KP, ATCC 4352) (Gram-negative). This was done to check the antimicrobial efficacy of different zinc nanocomposite fabrics against various pathogens under different conditions.

#### Before-wash antimicrobial test

**Figure 5:**
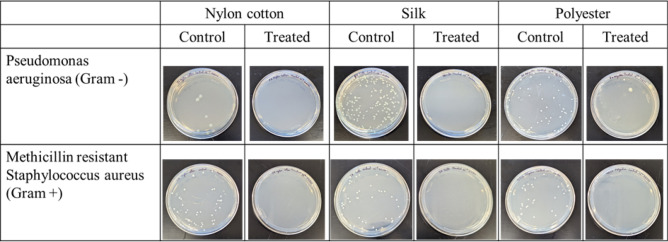
Selected photos of cell culture plates used in antimicrobial testing via cell counting for samples eluted after 24 h with 10^5^X dilution.

Briefly, the fabric samples were inoculated with suspensions of bacteria in nutrient broth and rinsed. These samples were divided into two sets with different elution times, a 0-h immediate elution and an elution after a 24-h incubation period. The eluted solution from the inoculated fabrics was then plated and incubated for 24 h at 37 °C. Bacterial growth was quantified through colony counting on plates as pictured in Fig. 5. The results are reported in % reduction and calculated by the following formula (Eq. 1).

1 $$100{*}\frac{{\left( {B - A} \right)}}{B} = {\text{\% }}Reduction$$

where **A** is the number of bacterial colonies recovered from the inoculated treated sample fabric and **B** is the number of bacterial colonies recovered from the inoculated untreated control fabric, both incubated over a set contact period. For all experiments, Eq. 2 was used to determine test efficacy. This calculation is a qualitative check to confirm that the initial concentration of bacteria used was enough to do the antimicrobial test. This number must be higher than 1.5.

2 $$\log \left( b \right) - \log \left( a \right) > 1.5$$

where **a** is number of bacterial colonies recovered from untreated control fabric immediately after inoculation and **b** is the number of bacterial colonies recovered from untreated control fabric after 24-h incubation post inoculation. For all reported experiments, these efficacy values ranged from 1.5 to 3.5, confirming the effective bacterial concentration.

The above procedure was followed similarly for tests against the fungal pathogen *Candida albicans.* Although AATCC recommended antifungal tests for textiles differ from that of bacteria, the growth cycle of *Candida albicans* resembles that of bacteria, which enables antifungal testing using this modified version of the AATCC Test Method 100–2004^35^.

The 0-h data, shown in Table 1, for each textile showed varied antibacterial properties for *Pseudomonas aeruginosa (PA),* methicillin resistant *Staphylococcus aureus (MRSA)* and *Candida albicans (CA)*. This could be due to the quick elution of microbial inoculations from the fabrics, leaving much less time for the nanoparticles to interact with the pathogenic cells and reduce their growth consistently. Despite this, the microbial reduction percentage for almost all samples ranged from 32 to 91% indicating that the nanocomposite fabrics can still induce some antimicrobial properties. For nanocomposite polyester treated with MRSA, negative value was observed and indicate that there were more bacteria recovered than control. This could be possible because of variable absorption properties of each fabric or might be because of improper mixing of bacterial concentration. “NA” is used for either negative value or indicates contamination. The 24-h elution results on the other hand showed significant microbial reduction in samples treated with zinc nanocomposite textiles compared to untreated controls (ANOVA, *p* < 0.05). The microbial reduction percentages were far less variable and ranged from 98 to > 99.999% (> 1 log10 to > 5 log10) reduction. The percentages obtained are higher than or similar to those reported in the literature for antimicrobial cotton fabrics against Gram-positive *S. aureus* and Gram-negative bacteria^36,37^. Though the antimicrobial mechanism of zinc nanoparticles is not clearly studied, reports from previous studies have suggested that their photocatalytic properties generate hydrogen peroxide (H_2_O_2_) through a series of reactions in the presence of H_2_O. These hydrogen peroxide molecules are toxic to the microbial cells as they can penetrate the cell membrane and kill them^38^. There have been fewer studies on the antimicrobial efficacy of these textiles on fungal pathogens. However, the results obtained here indicate that these novel nanocomposite textiles can also be used for antifungal applications.

**Table 1:** Microbial reduction (%) of *Pseudomonas aeruginosa (PA),* methicillin resistant *Staphylococcus aureus (MRSA)* and *Candida albicans (CA)* after 0 h of incubation (immediate elution) and after 24 h with zinc nanocomposite textiles (The zinc nanocomposite textiles were washed once as part of the manufacturing process to remove loose particles; microbial reduction in terms of log reductions are provided for some of the samples in brackets).

Sample	% Reduction after 0 h			% Reduction after 24 h		
	PA	MRSA	CA	PA	MRSA	CA
Nylon cotton	72.5	47.85	55.63	> 99.9999 (> 6 log10)	> 99.999 (> 5 log10)	> 99.99 (> 4 log10)
Silk	32.5	90.39 (> 1 log10)	46.3	> 99.9999 (> 6 log10)	99.9999 (6 log10)	> 99.999 (> 5 log10)
Polyester	75	NA	60.32	98.35 (> 1 log10)	99.9999 (6 log10)	> 99.999 (> 5 log10)

#### After-wash antibacterial test

##### Third-party wash durability analysis

Zinc nanocomposite polyester cotton was machine washed 50 times per AATCC LP1: Home Laundering method and the antibacterial properties were assessed separately by a third-party testing through Vartest Laboratories LLC. The bacterial reduction was found to be 0% for both *Klebsiella pneumoniae (KP,* Gram-negative) and *Staphylococcus aureus (SA,* Gram-positive) indicating that the nanocomposite textile did not exhibit antimicrobial properties during the 0-h (immediate elution) process after 50 wash cycles (See Table 2 and supplementary information). However, the same material exhibited antimicrobial properties even after 50 wash cycles with more than 99.999% (5 log10) bacterial reduction for both *Klebsiella pneumoniae* and *Staphylococcus aureus* after 24-h incubation (See Table 2 and supplementary information)*.* While there have been some studies in the past on the application of zinc oxide nanoparticles for the fabrication of antimicrobial textiles, most of these show much lower microbial reduction percentages than the desired 99.999% (5 log10) range for samples washed over 20 cycles^39,40^. The zinc nanocomposite textiles prepared using the novel Crescoating technique on the other hand could retain their antimicrobial properties even after being reused over 50 times repeatedly. Preliminary tests on samples washed for 100 cycles (see next section) also show promising antibacterial activity. Therefore, these fabrics can be successfully applied to manufacture antimicrobial textiles in the healthcare industry.

**Table 2:** Microbial reduction (%) of *Klebsiella pneumoniae (KP)* and *Staphylococcus aureus (SA)* after 0 h of incubation (immediate elution) and after 24 h with zinc nanocomposite textiles subjected to 50 wash cycles. Results obtained from third party independent testing (Independent evaluation conducted by Vartest Laboratories LLC).

Sample	% Reduction after 0 h		% Reduction after 24 h	
	KP	SA	KP	SA
Polycotton	0	0	> 99.999 (> 5 log10)	> 99.999 (> 5 log10)

##### Laboratory wash durability analysis

Zinc nanocomposite cotton fabric was washed 100 times by following the AATCC LP1: Home Laundering method. These samples were then tested against the Gram-positive *Staphylococcus aureus* bacteria according to AATCC test method 100. The results from the 24-h elution samples show > 99.999% (> 5 log10) reduction indicating that the nanocomposite fabrics can retain their antimicrobial behavior even after several washes. This means that the novel nanocomposite fabrics have better longevity and durability when compared to other antimicrobial textiles previously studied^39,40^.

### Safety and skin irritation test

To determine whether the antimicrobial nanocomposite fabrics were safe to use as clothing, the Human Repeat Insult Patch Test (HRIPT) was conducted by Evalulabs LLC. The tests were conducted after obtaining informed consent from 50 human subjects and was carried out under the supervision of a licensed dermatologist. The protocol was approved by the Ethics Committee and the Evalulab LLC Independent Ethics Committee (IEC) prior to the tests to protect the rights of the human participants and the tests were performed in accordance with the relevant guidelines and regulations. The results indicated that the fabrics were non-irritating and hypoallergenic to human skin. The full report of this test is available in the supplementary information.

## Materials and methods

### Nanocomposite synthesis

Zinc acetate dihydrate (Sigma Aldrich, USA) was used as a precursor. Textile fabric supports used were polyester cotton, nylon cotton (50/50 nylon cotton camouflage ripstop fabric) (Rockywoods Fabrics LLC), silk adjacent fabric, 60 g/m^2^ (ISO 105-F06, Testfabrics Inc.,) and synthetic polyester adjacent fabric, 130 g/m^2^ (ISO 105-F04, Testfabrics Inc.). For synthesis on zinc nanocomposite textiles, solutions of zinc salts were prepared in filtered deionized water with ~ 18 megohm cm^-1^ conductivity (SpectraPure, USA). In a typical synthesis process, fabrics were submerged in the solutions for 30 min at room temperature. Next, the fabrics were heated at 100 °C in a convection oven (Model FDL 115, BINDER GmbH, Tuttlingen, Germany) for 4 h.

### Bacterial culture

The bacterial strains used for testing were the Gram-positive *Staphylococcus aureus subsp. aureus* (ATCC 6538) and methicillin resistant *Staphylococcus aureus*, the Gram-negative *Pseudomonas aeruginosa* (ATCC 27,853) and the fungus *Candida albicans.* All organisms were cultured in tryptic soy broth (TSB) followed by serial dilution to reach a final concentration of 10^5^ CFU/ml for fabric inoculation. The concentration was confirmed by plate counting method prepared in triplicates. The Gram-positive *Staphylococcus aureus subsp. aureus* (ATCC 6538) and the Gram-negative *Klebsiella pneumoniae* (ATCC 4352) were used for third party tests.

### Quantitative antimicrobial tests

Antimicrobial performance was quantified using a modified form of the plate counting method outlined in AATCC Test Method 100–2004. The materials tested for antimicrobial activity were zinc nanocomposite textiles, which include silk, nylon cotton, and polyester (PE) fabrics. The tests were conducted on swatches that have been machine washed once after fabrication, considered as “before washing” samples and those that were subjected to machine washing and drying cycles considered as “after washing” samples. The controls were untreated fabrics. One swatch of untreated fabric and one swatch of treated fabric were each placed in separate 60 mm × 15 mm petri dishes. The swatches were uniformly cut to the measurement of 4 X 2.5 cm. The untreated and treated fabrics were then inoculated with a concentration 10^5^ CFU/ml of each microbial strain as prepared previously. The purpose of this inoculation is to completely soak the swatch of fabric with the culture. The petri dishes were then sealed with parafilm and incubated for 24 h at 37 °C. For 0-h immediate elution tests, samples were prepared similarly and immediately transferred to tubes containing 5 mL of Hank’s Balanced Salt Solution (HBSS buffer). The microbial culture was then completely eluted from the swatches via thorough vortexing. Following this, the swatches were removed from the HBSS buffer solution. The 24-h incubated samples were similarly eluted. One tube of 5 mL of HBSS solution was directly inoculated with the microbial culture and served as a control. All the eluted samples were serially diluted using HBSS, thrice for immediate elution samples and five times for 24-h elution samples and plated on tryptic soy agar (TSA) followed by incubation at 37 °C for 18–24 h.

The number of microbial colonies were counted after incubation and the reduction percentage was calculated as described previously.

### Textile laundering

For further laundering tests, nanocomposite polyester cotton textiles were subjected to washing cycles per AATCC LP1: Home Laundering method in a machine washer (Vortex M6, SDL Atlas) followed by drying cycles in a tumble dryer (Vortex M6D, SDL Atlas). This method involves a 16 min wash cycle with warm water at high agitation followed by rinsing for 2 min 30 s, spinning for 5 min and finally high heat tumble drying cycles. The total load weight of the fabrics was 1.8 ± 0.1 kg and the water level was 72 ± 4 L. AATCC High Efficiency Liquid Standard Reference detergent was used for all washing steps. The samples were subjected to 50 and 100 wash cycles to test their durability and longevity.

### Statistical analysis

The antimicrobial tests were conducted in triplicates for both 0-h and 24-h contact periods. Geometric means and standard deviations were calculated and used for the statistical analysis. One-way ANOVA was used to do the analysis of variance and the significant differences in the means were tested at significance = 0.05.

## Conclusion

A novel Crescoating technology has been applied as a promising technique to synthesize highly efficient antimicrobial nanocomposite fabrics. Antimicrobial tests conducted on zinc nanocomposite textiles against Gram-positive and Gram-negative bacteria and fungi showed > 99.999% (> 5 log10) microbial reduction. The fabrics are also safe, highly durable and can be reused over 100 wash/dry cycles without loss of their functionality. Third party dermatological tests showed that the nanocomposite fabric materials are non-irritating and hypoallergenic to human skin. Therefore, these fabrics can be successfully used as medical textiles such as hospital linen and surgical gowns, which can aid in the fight against nosocomial infections and disease transmission in healthcare setting.

## Supplementary Information

Supplementary Information.

## Data Availability

The datasets generated during and/or analyzed during the current study are available from the corresponding author on reasonable request.
