# Supplementary material for: Highly efficient and durable antimicrobial nanocomposite textiles
Source: Sci Rep. 2022 Oct 15;12:17332. doi: 10.1038/s41598-022-22370-2 (PMC9568944; doi:10.1038/s41598-022-22370-2)

Highly Efficient and Durable Antimicrobial Nanocomposite Textiles

*Vinni Thekkudan Novi^1^, Andrew Gonzalez^2^, John Brockgreitens^2^, Abdennour Abbas^1,2^**

^1^ Department of Bioproducts and Biosystems Engineering

University of Minnesota-Twin Cities

2004 Folwell Ave

St. Paul, MN 55108, USA

^2^ Claros Technologies Inc.

1000 Westgate Drive

Suite 1005

St. Paul, MN 55114, USA

Corresponding author: Dr. Abdennour Abbas

Email: [aabbas@umn.edu](mailto:aabbas@umn.edu), dennor@clarostech.com

Phone #: +1 (612) 624-4292

**Supplementary Information**


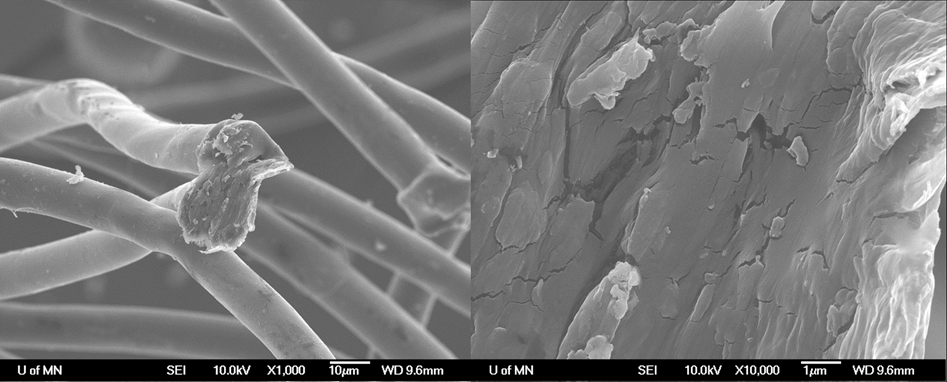


**Supplementary Figure 1:** SEM images of untreated polyester control fabric

**Supplementary Table S1:** Synthetic Precipitate Leachate Procedure results from Pace Analytical for three types of cotton samples. The samples were washed 1, 50 and 100 times after functionalization.

| Zinc Discharge (µg/L) | | | | | | | | | |
| --- | --- | --- | --- | --- | --- | --- | --- | --- | --- |
|  | # of Washes | | | | | | | | |
|  | 1 | | | 50 | | | 100 | | |
| Cot #1 | 5030 | 5610 | 4680 | 543 | 575 | 366 | 1150 | 848 | 1160 |
| Cot #2 | 12800 | 11100 | 9350 | 519 | 371 | 352 | 571 | 558 | 544 |
| Cot #3 | 57100 | 106000 | N/A | 1500 | 1180 | 1930 | 1180 | 1140 | 961 |


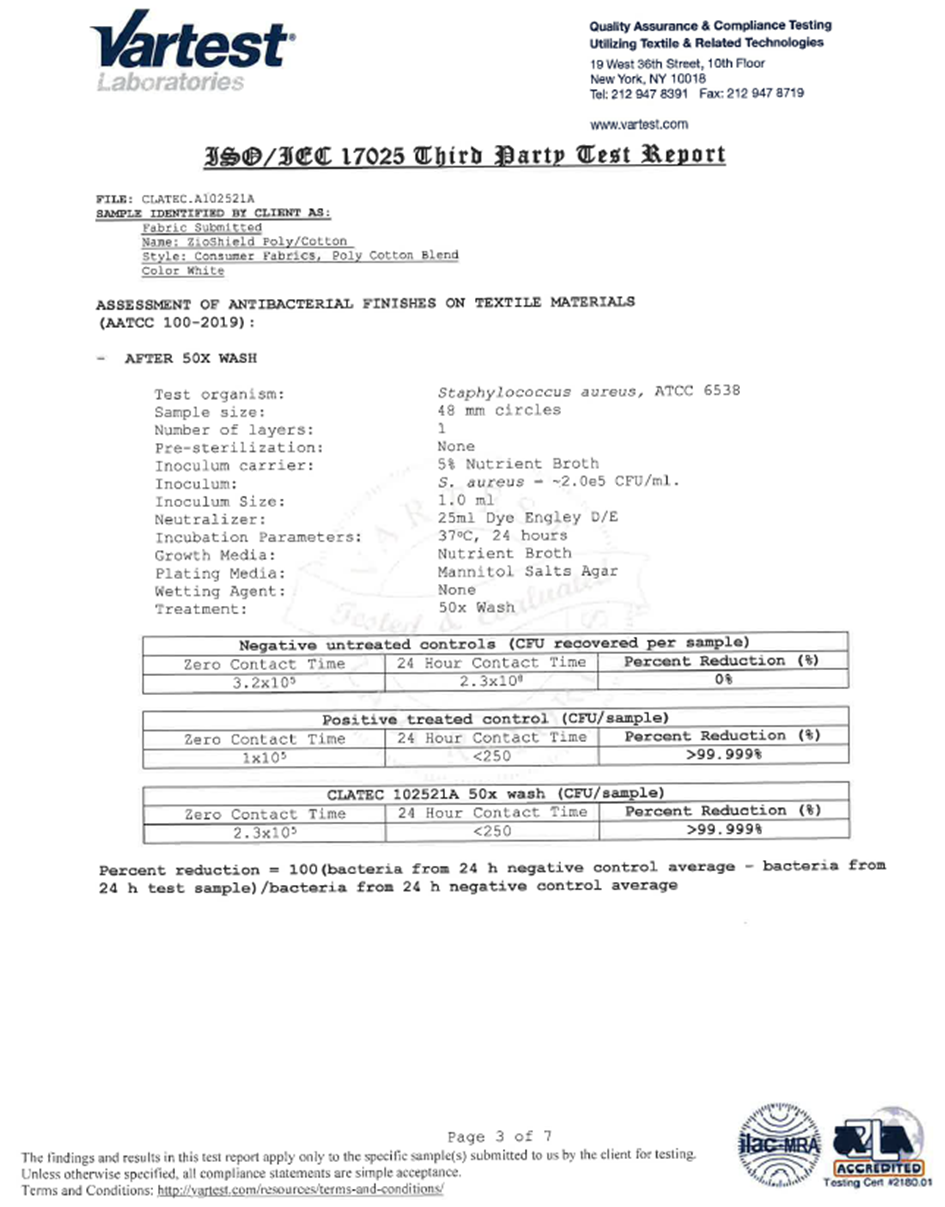


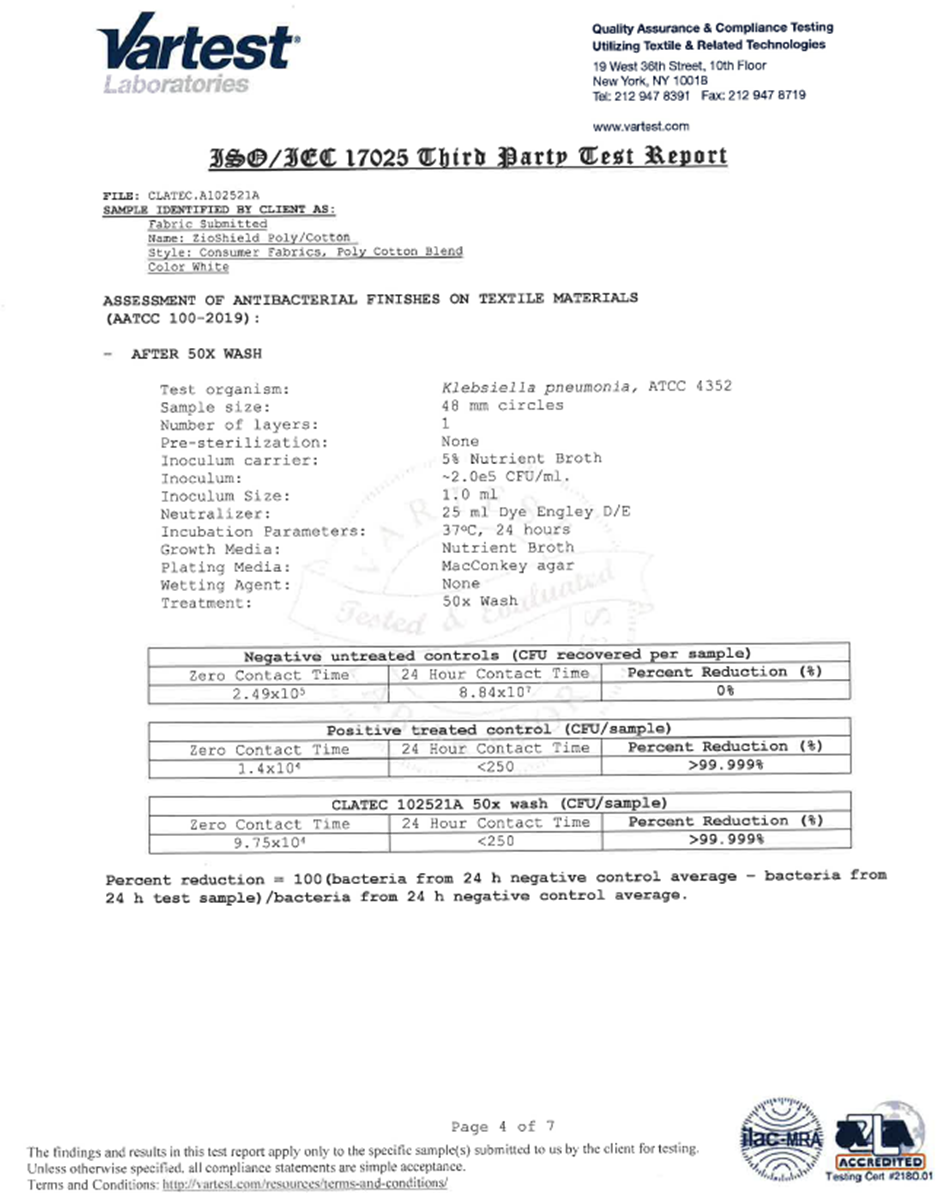


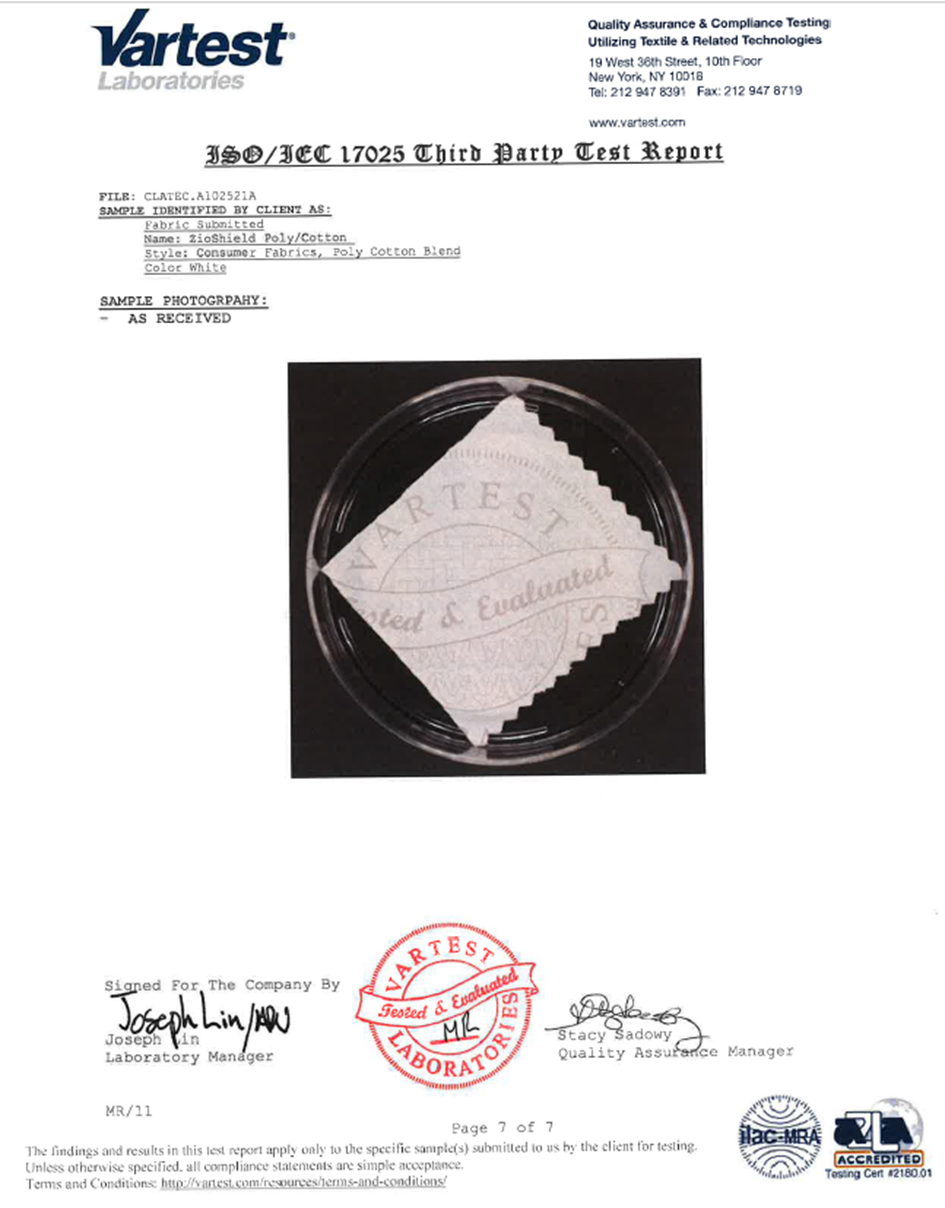


Parts of the antimicrobial test results from the third-party testing company Vartest Laboratories LLC is displayed here. The full report is available upon request.


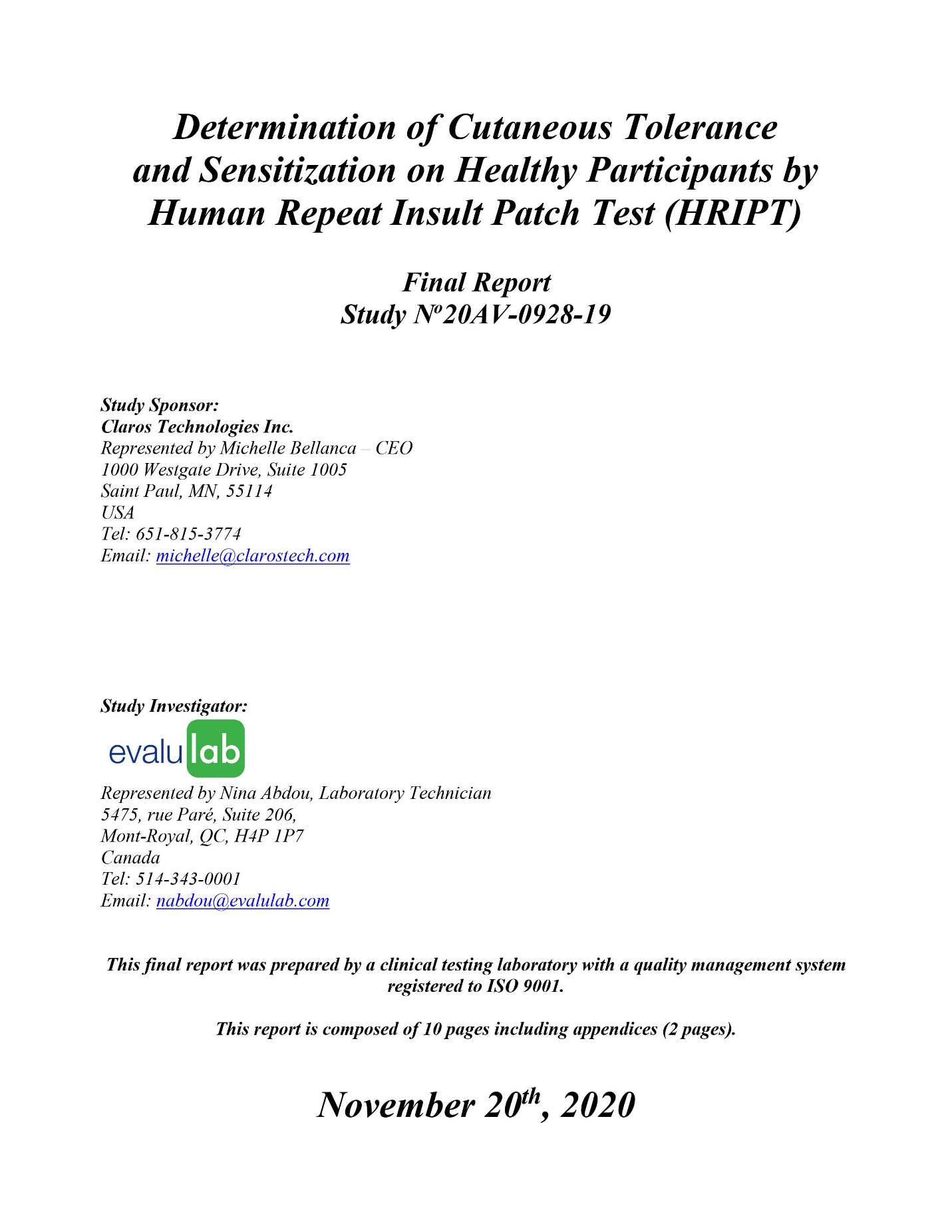


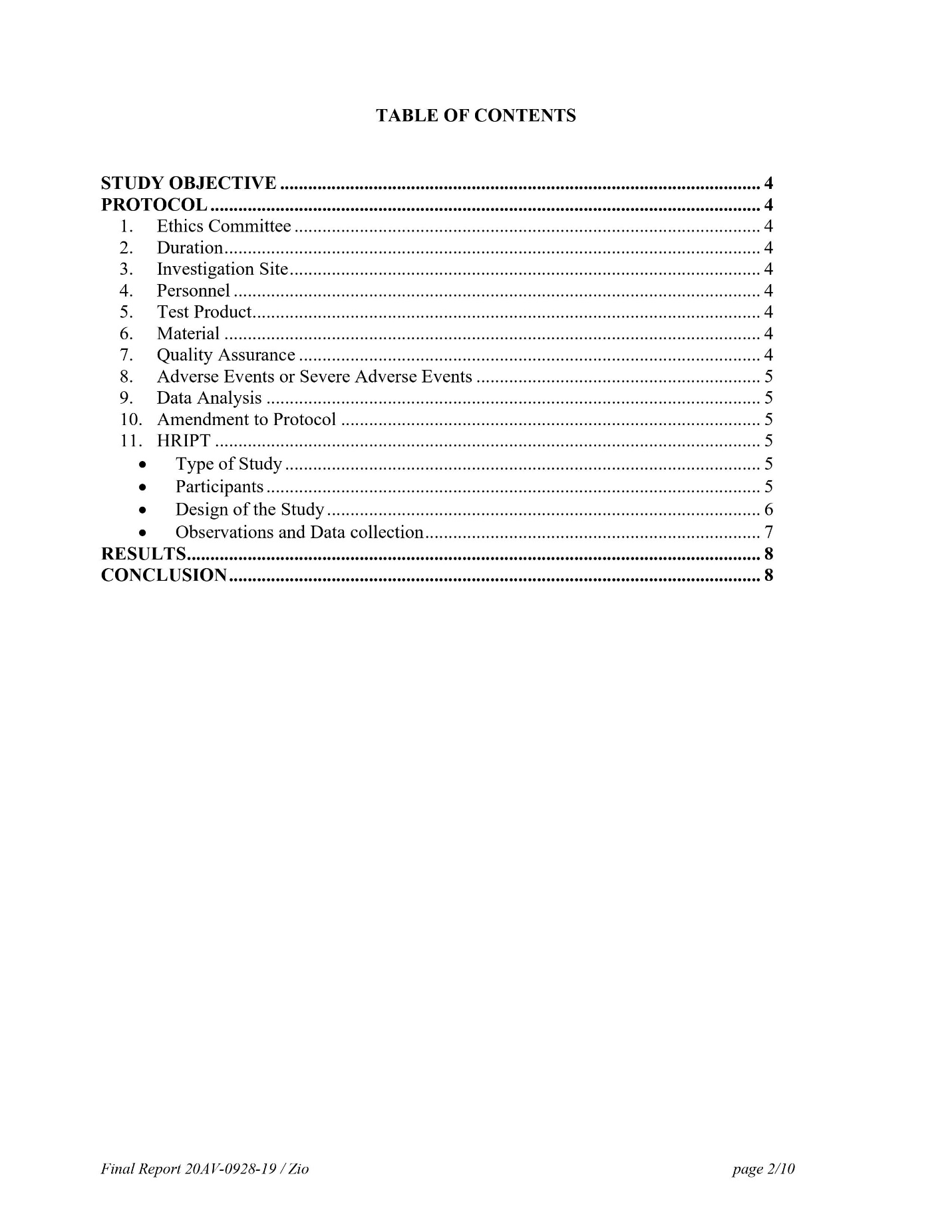


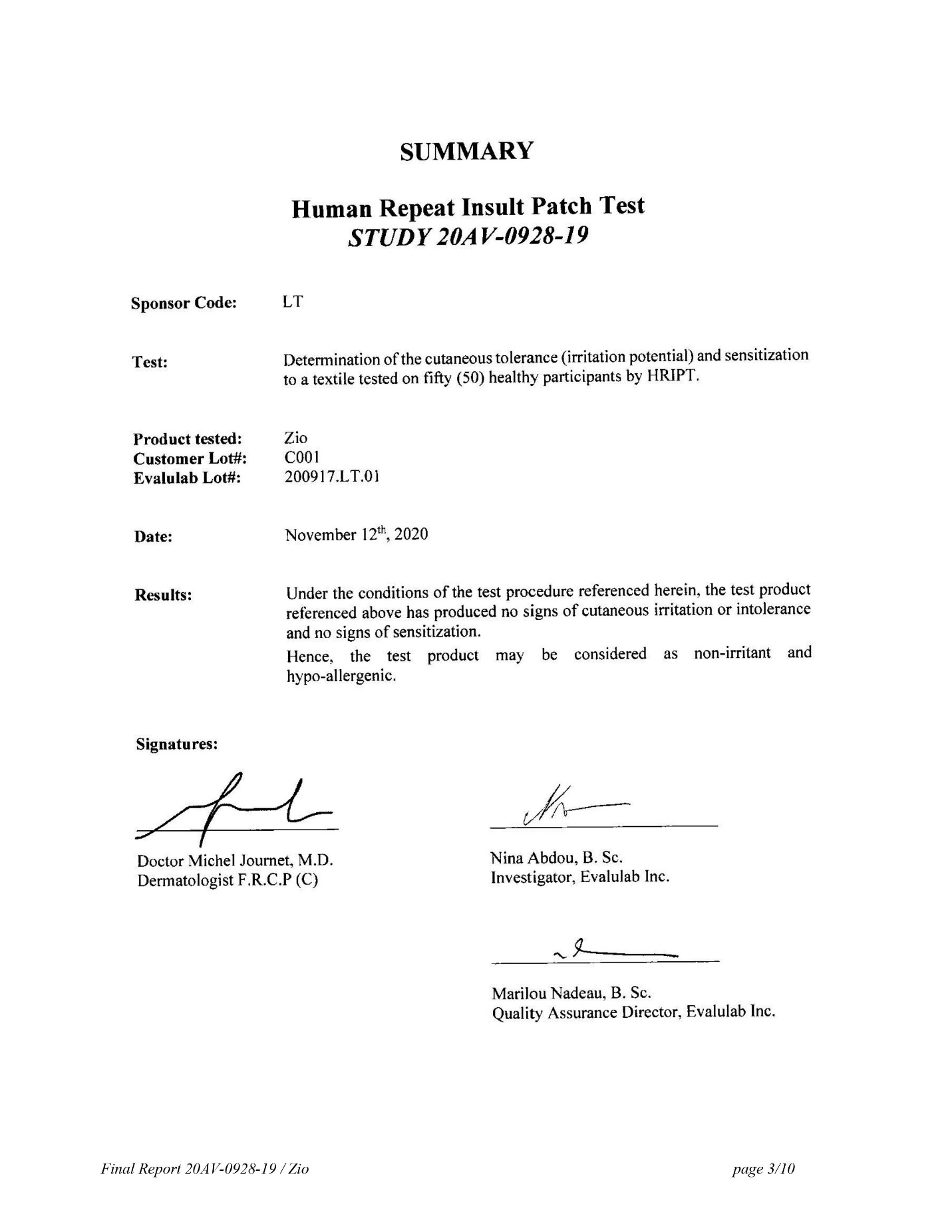


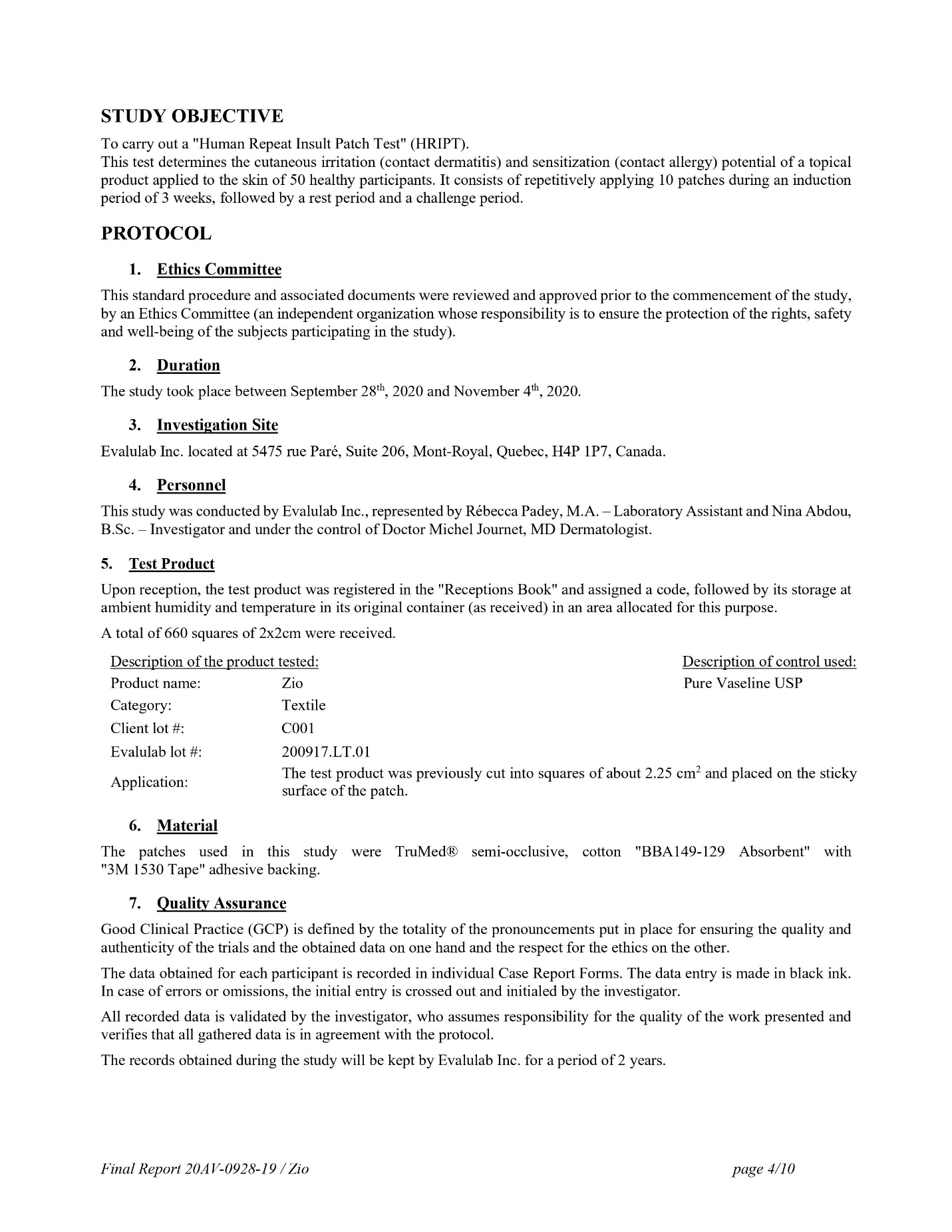


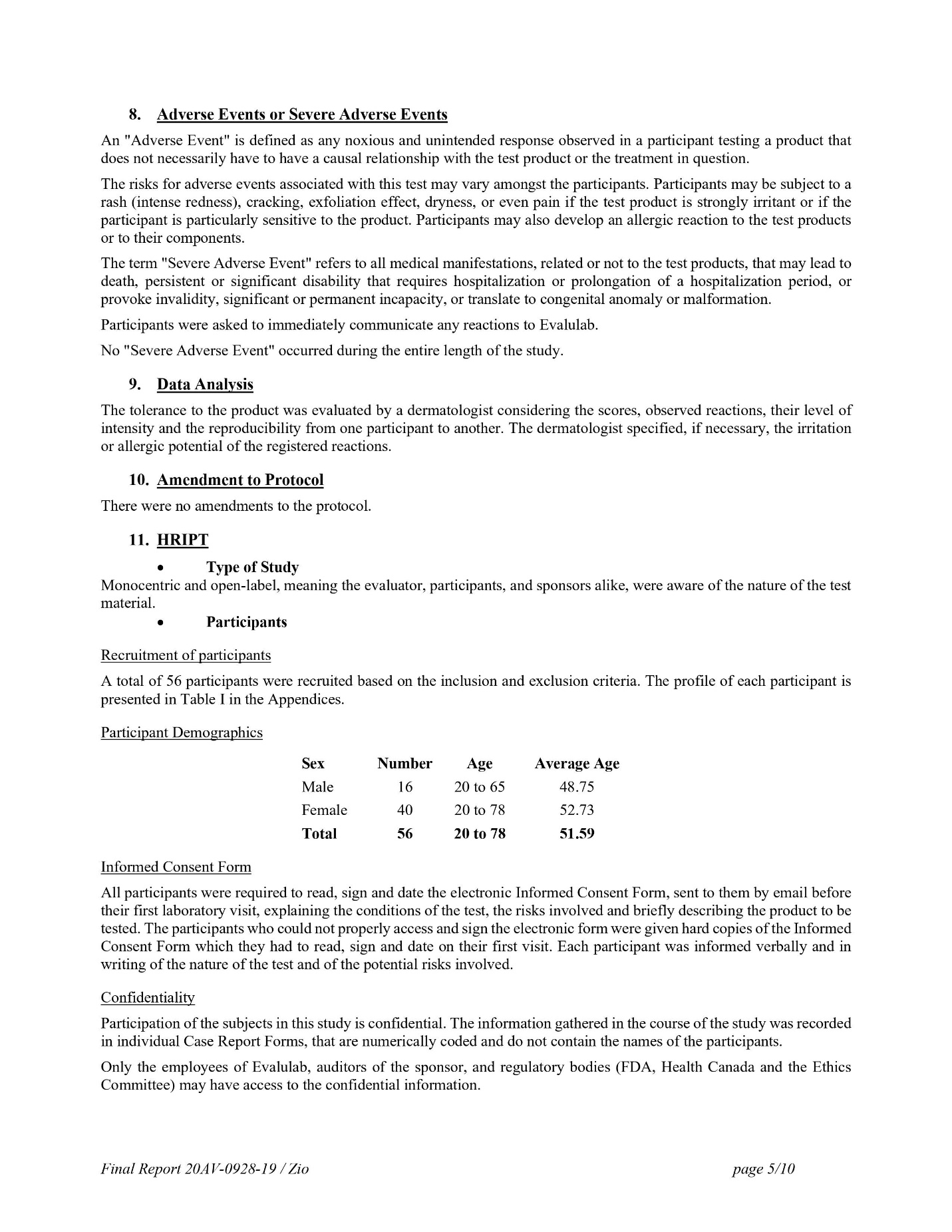


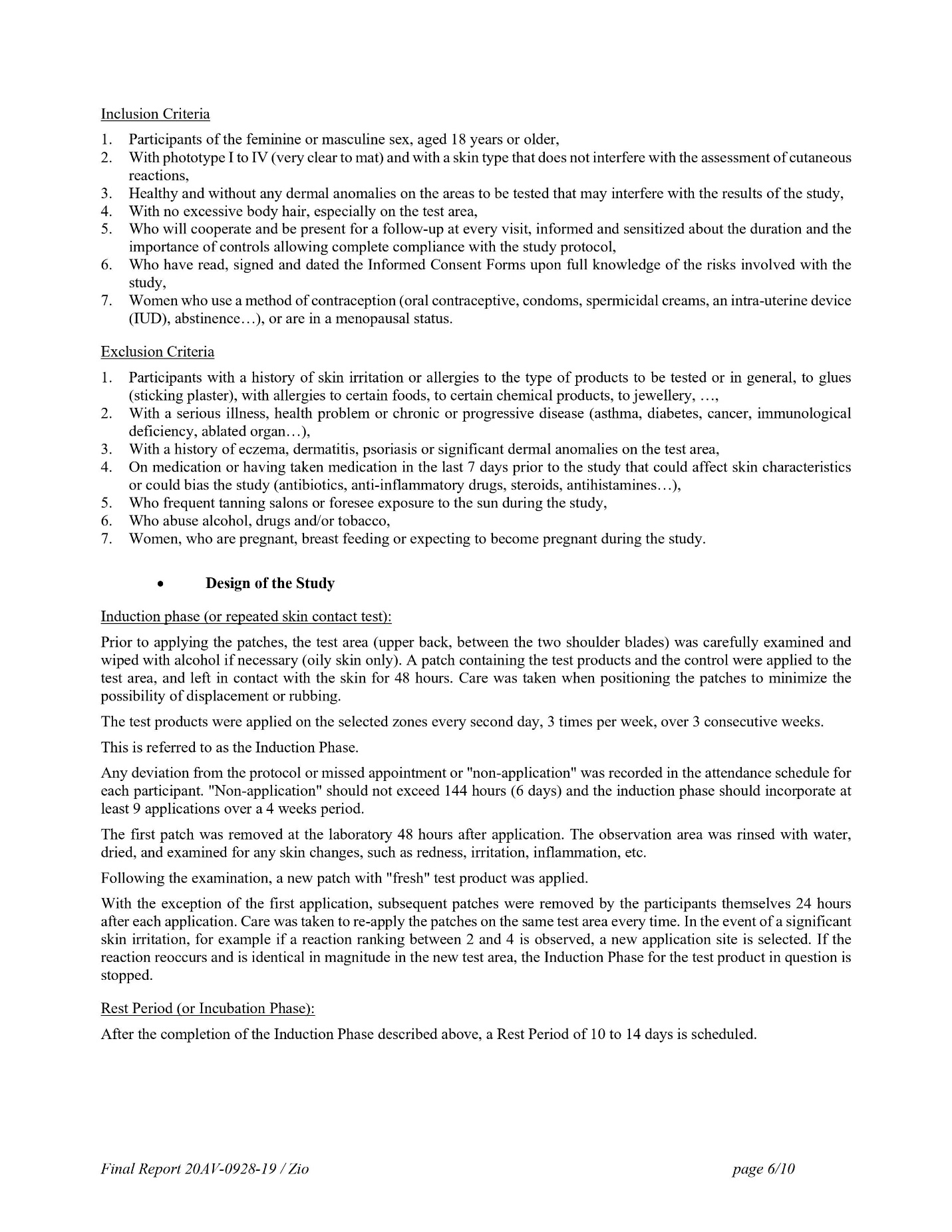


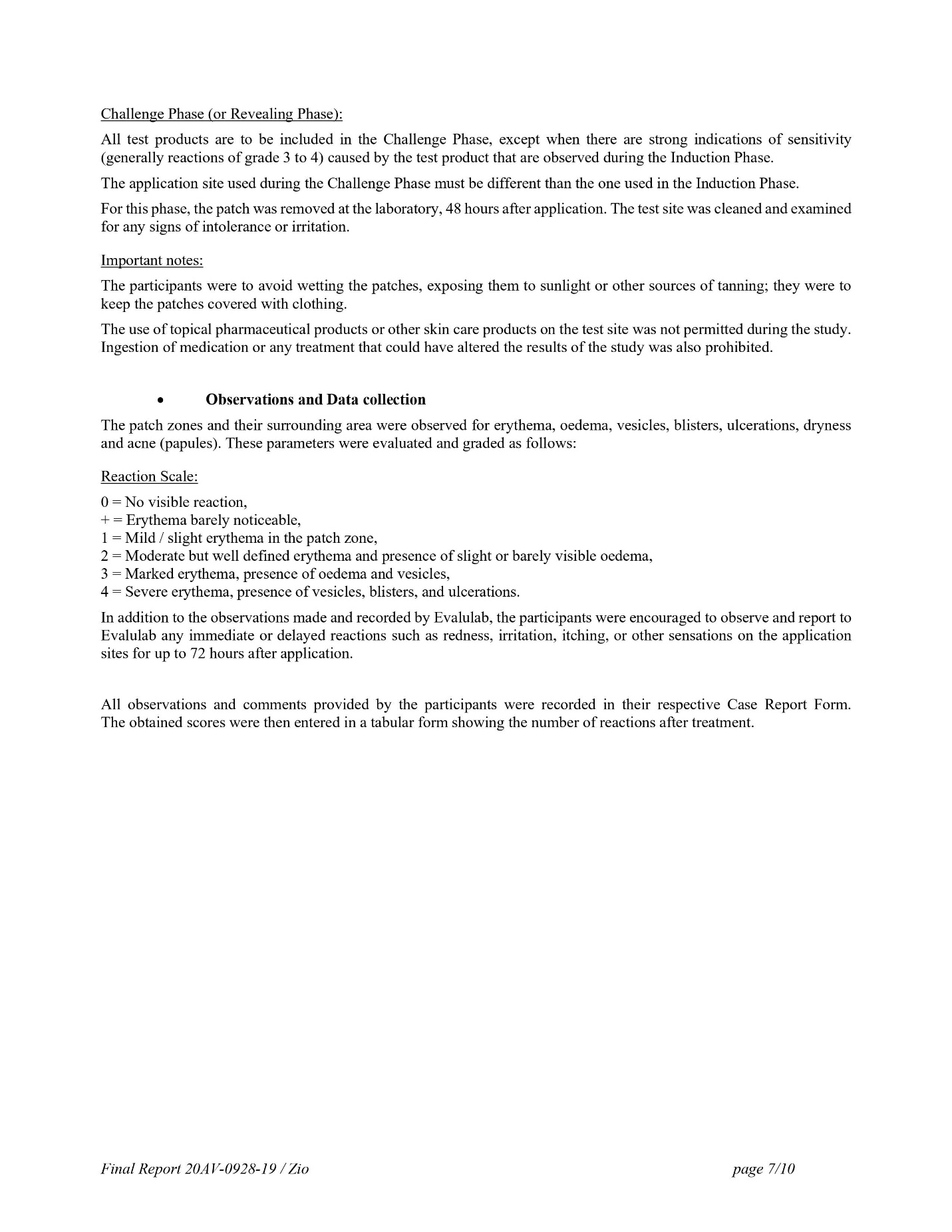


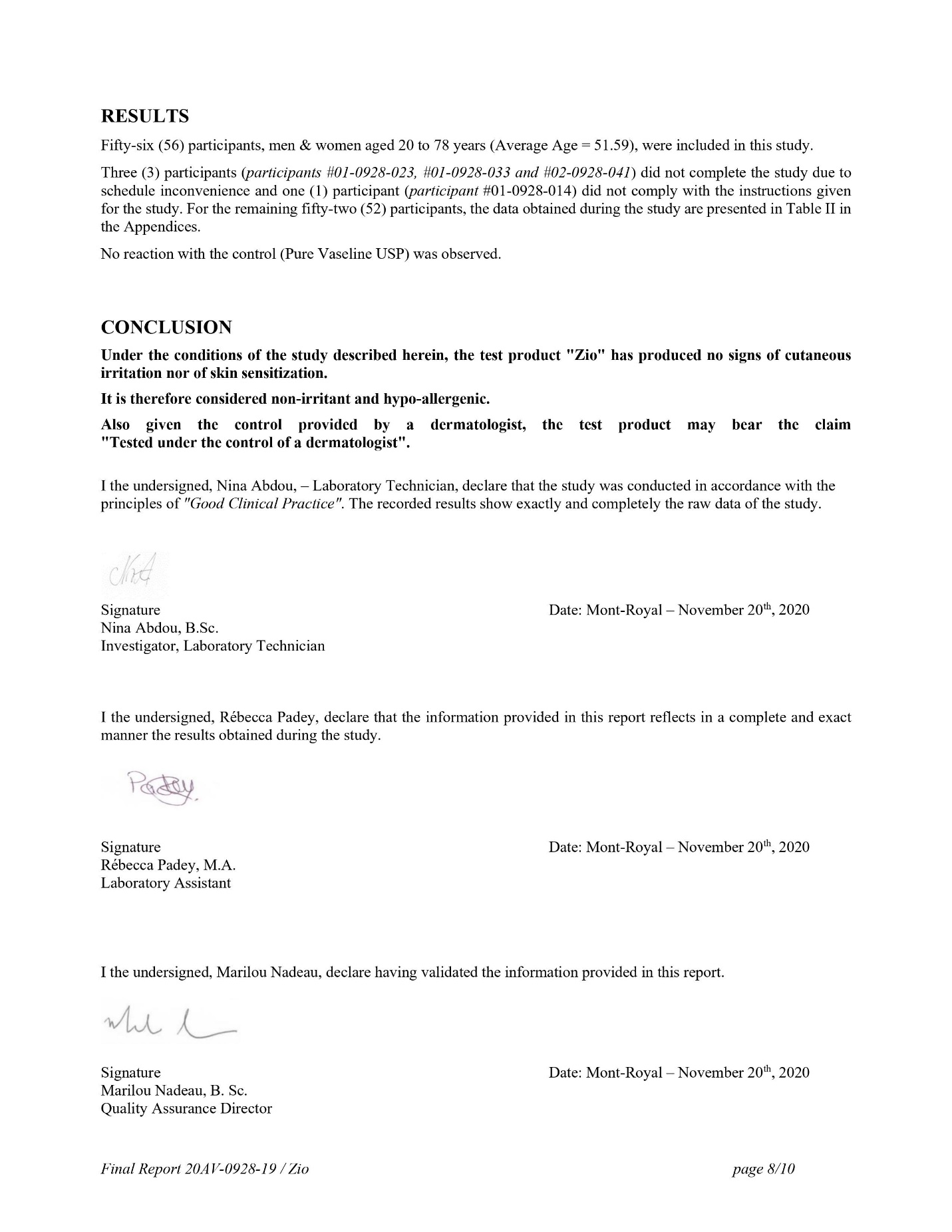


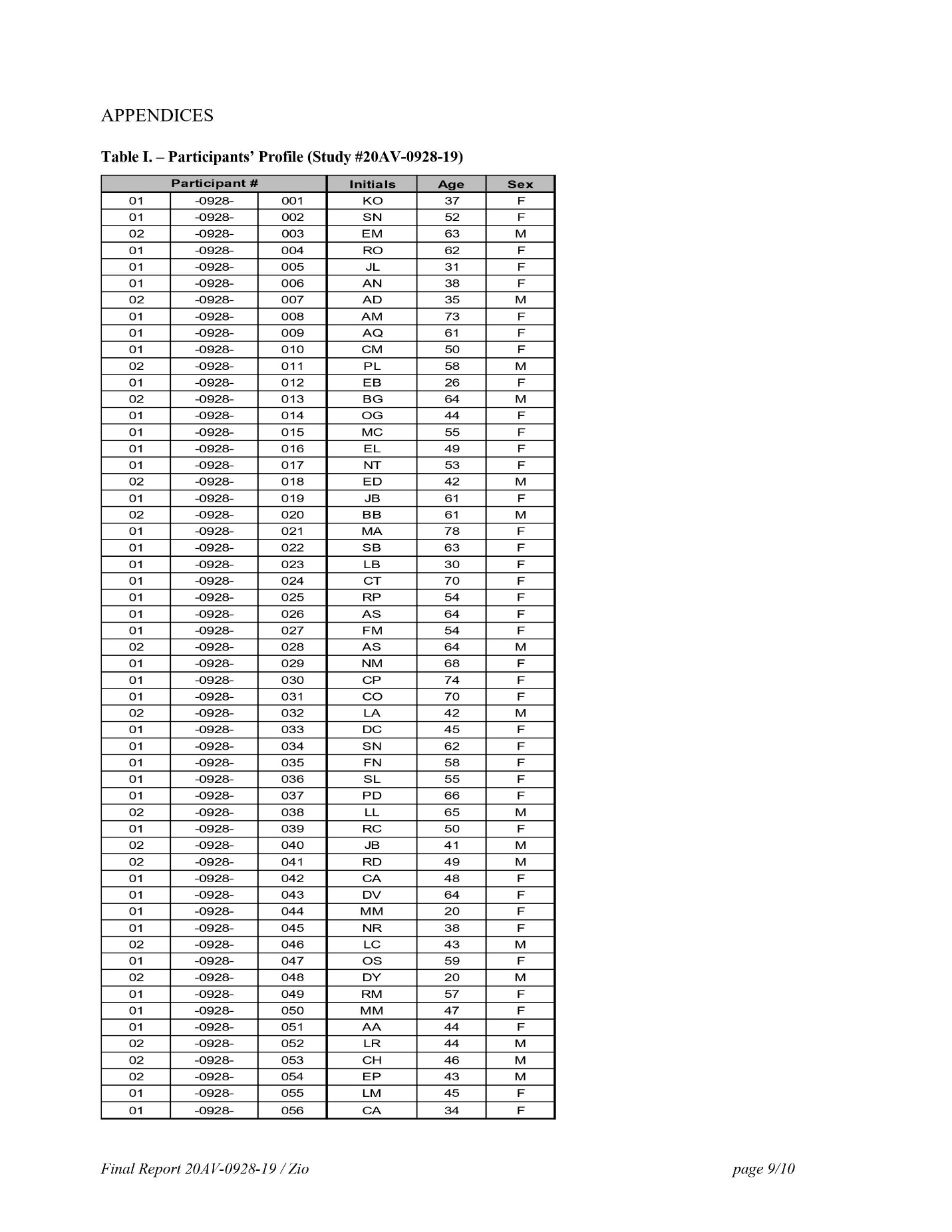


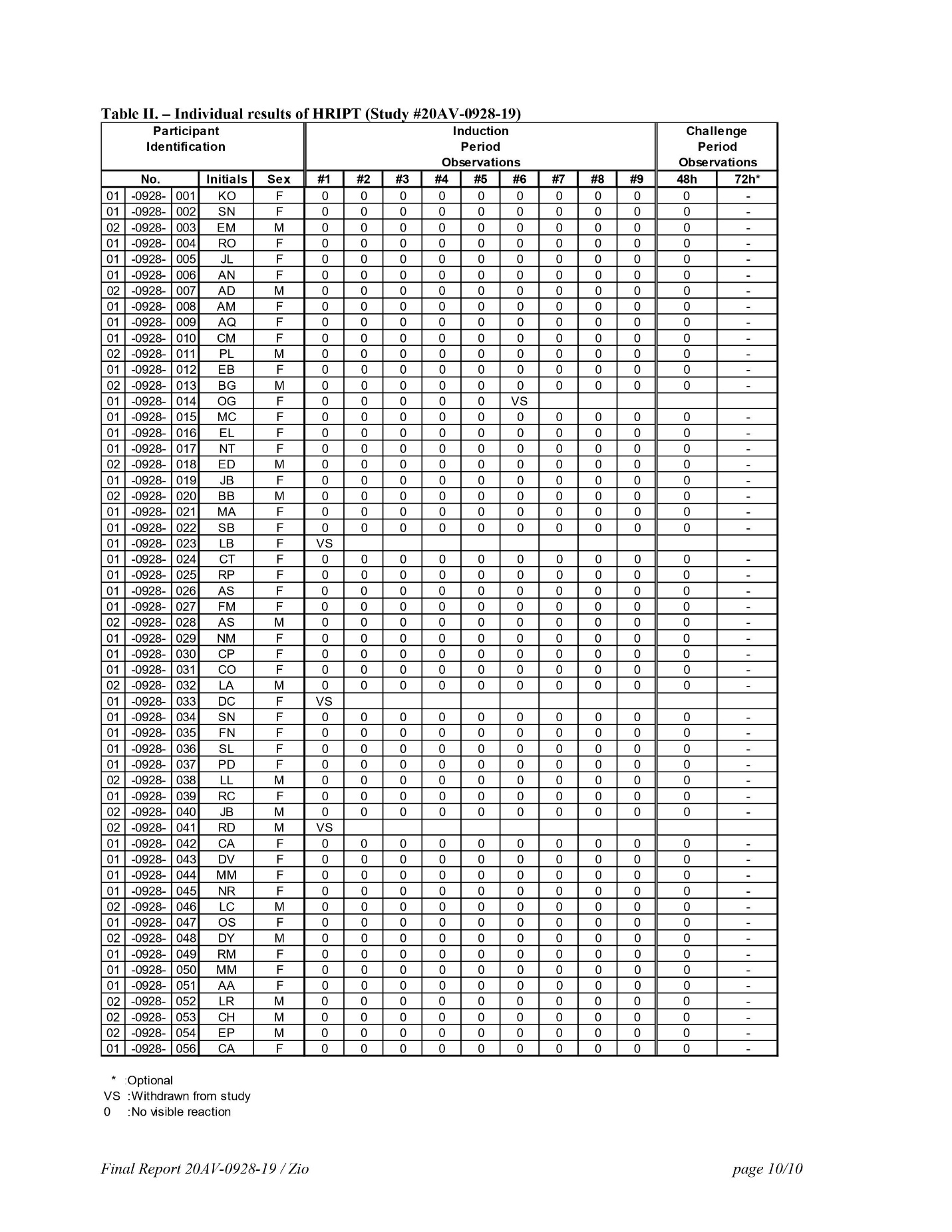

Supplement: Supplementary file 1 — Supplementary Information. [file 41598_2022_22370_MOESM1_ESM.docx]
